# Paraduodenal hernia complicated with intussusception: case report

**DOI:** 10.1186/s12893-018-0460-x

**Published:** 2018-12-22

**Authors:** Chong Jin, Jinggang Mo, Guoyu Wang, Hao Jiang, Yifu Feng, Song Wang

**Affiliations:** 1 Department of General Surgery, Taizhou Central Hospital, Taizhou University Hospital, Taizhou, 318000 China; 2 Department of Radiology, Taizhou Central Hospital, Taizhou University Hospital, Taizhou, 318000 China

**Keywords:** Paraduodenal hernia, Intussusception, Internal hernia

## Abstract

The diagnosis of paraduodenal hernia is still a challenge in clinical practice due to lacking of specific symptoms. Case presentation: An 83-yr-old male patient presented to our department due to severe abdominal pain for 8 h. Abdominal contrast enhanced computerized tomography (CT) scan indicated intussusception in the duodenum and the upper segment of jejunum, as well as internal hernia. He complaint of progression in the abdominal pain, and then laparoscope was carried out, which indicated left-sided paraduodenal hernia. Subsequently, the patient was transferred to celiotomy, during which slight ischemic changes were noticed in the intestinal canal. Meanwhile, a hernial orifice was noticed in the left orifice of the duodenum. Conclusions: In this case, we presented our experiences on the diagnosis of paraduodenal hernia and intussusception. Our study contributed to the understanding, early diagnosis and selection of surgical options for the surgeons.

## Background

Paraduodenal hernias, a type of internal abdominal hernima associated with the pathogenesis of intestinal obstruction, is frequently complicated by volvulus and ischemia [1, 2]. The diagnosis of paraduodenal hernias is still a challenge due to lacking of specific clinical findings as most patients usually show abdominal pain and distention, nausea or vomiting. With the disease progression, a large number of cases may present intestinal canal necrosis, intestinal perforation and even death [3, 4].

To date, different diagnostic imaging techniques have been utilized for the diagnosis of paraduodenal hernias and the complications [5]. To our best knowledge, rare reports on paranuodenal hernias complicated with intussusception are available. In this case, we presented a rare case of paranuodenal hernias complicated with intussusception.

## Case presentation

**Fig. 1:**
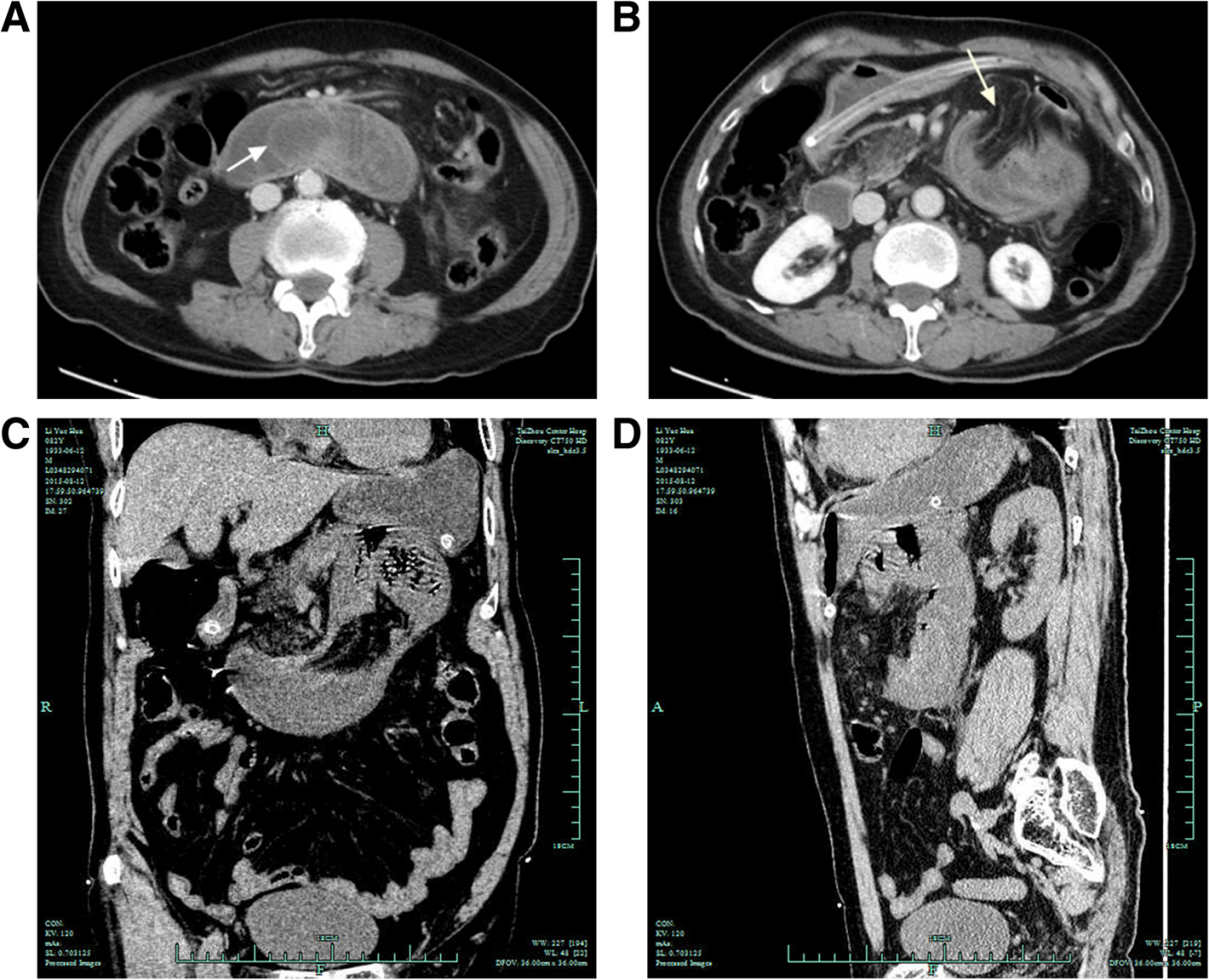
The patient showed typical target-ring signs (white arrow) with part of the jejunum enclosed with the horizontal segment of the duodenum (**a**). Aberrant condensation was noticed in the small intestine in the left upper abdomen. The upper segment enclosed with the jejunum paraduodenal recess, which was represented by sac structures (white arrow, **b**). **c**, **d** Coronal and sagittal findings of CT

**Fig. 2:**
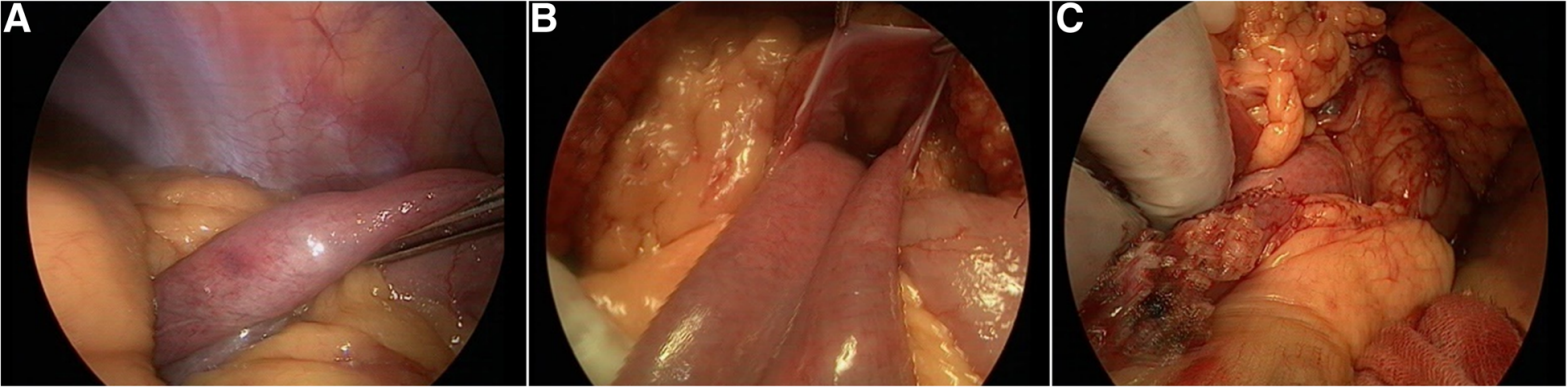
Laparoscopic exploration indicated entry of jejunum into the paraduodenal recess (**a**). Hernia orifice in left orifice of the duodenum (**b**). Findings after hernial orifice cut (**c**)

An 83-yr-old male patient presented to our department due to severe abdominal pain for 8 h, especially the peripheral umbilicus. The pain showed no obvious attention when changing the body position. On physical examination, no tenderness or rebound tenderness was felt in the peripheral umbilicus. Abdominal contrast enhanced CT scan indicated intussusception in the duodenum and the upper segment of jejunum, as well as internal hernia (Fig. 1). He received no abdominal surgery before. Besides, he complaint of progression in the abdominal pain, and then laparoscope was carried out which showed left-sided paraduodenal hernia (Fig. 2a). The motion of the intestinal canal involved by hernia was poor. Subsequently, the patient was transferred to celiotomy, during which slight ischemic changes were noticed in the intestinal canal. A hernial orifice was noticed in the left orifice of the duodenum (Fig. 2b). No obvious intestinal necrosis was identified, the hernial sac wall was resected and the orifice was completely cut (Fig. 2c). The patient was followed up for 2 two years until now with no recurrence. Written informed consent was obtained from the patient. The study protocols were approved by the Ethical Committee of the Taizhou Central Hospital.

## Discussion and conclusions

Intussusception occurs when a segment of bowel telescopes into an adjacent segment. Primary intussusception is common in the intestinal canal in children with no pathological changes, while the secondary intussusception is usually present in adults with intestinal disorders such as tumor, polypus, tubercle, adhesion and Meckel diverticulum. In this case, the intussusception was induced by paraduodenal hernia.

Paraduodenal hernia is associated with the entry of abdominal organs into the paraduodenal recess. According to the relative position of the recess and the ascending part of duodenum, it is divided into two types including Landzert hernia and Waldeyer hernia with a ratio of 3:1 in prevalence [6]. To our best knowledge, most of the patients with paraduodenal hernia were manifested by intermittent abdominal pain and distention, and dyspepsia. In the presence of elevation of intra-abdominal pressure, the organs in the abdominal cavity, especially the small intestine, may enter the paraduodenal recess, which then induce intestinal obstruction features (e.g. abdominal pain and distention, nausea, and vomiting). However, for a patient with severe abdominal pain with unspecific conditions for intussusception, CT scan is effective for the diagnosis [7] which is highly relied on the presence of cystic mass containing the dilated small intestine in the junction between duodenum and jejunum [6]. In this case, after occurrence of paraduodenal hernia, the jejunum entered the herinal sac, which triggered elevation of the pressure in sac that may affect the enteroscinesia and the embolia of jejunum into the duodenum. Through the literature research until now, there was only one case reported by Catalano et al. in 2004 [8], in which a case of internal hernia with volvulus and intussusception was reported after presurgical CT scan. In our case, the patient showed typical target-ring signs. Besides, aberrant condensation was noticed in the upper segment of the jejunum, which entered into the paraduodenal recess. Moreover, the adjacent duodenum and posterior surface of stomach showed compression-induced shifting. The blood vessels in the mesentery of the hernial sac showed increase in number, as well as hyperemia and packing which converged into the hernia. Intraoperative exploration revealed a hernial sac in the left side of duodenum, combined with entry of jejunum, as well as dilatation and thickening in the horizontal segment of the duodenum. These confirmed the presence of paraduodenal hernia and intussusception.

Many patients with paraduodenal hernia may present incarcerated hernia, and may show risks of ischemia, gangrene and perforation in the absence of treatment options. Therefore, surgery is strongly recommended upon diagnosis. Nowadays, the diagnosis of paraduodenal hernia is still a challenge in clinical practice due to lacking of specific symptoms. In this case, we presented our experiences on the diagnosis of paraduodenal hernia and intussusception. Our study contributed to the understanding, early diagnosis and selection of surgical options for the surgeons.

## Abbreviations

CT: Computerized tomography
